# Mycorrhizal interactions do not influence plant–herbivore interactions in populations of *Clarkia xantiana* ssp. *xantiana* spanning from center to margin of the geographic range

**DOI:** 10.1002/ece3.4523

**Published:** 2018-10-26

**Authors:** Lana G. Bolin, John W. Benning, David A. Moeller

**Affiliations:** ^1^ Department of Plant and Microbial Biology University of Minnesota St. Paul Minnesota

**Keywords:** aboveground–belowground interactions, arbuscular mycorrhizal fungi, flowering phenology, geographic range, parasitism mutualism continuum, soil microbial community, tripartite multispecies interactions

## Abstract

Multispecies interactions can be important to the expression of phenotypes and in determining patterns of individual fitness in nature. Many plants engage in symbiosis with arbuscular mycorrhizal fungi (AMF), but the extent to which AMF modulate other species interactions remains poorly understood. We examined multispecies interactions among plants, AMF, and insect herbivores under drought stress using a greenhouse experiment and herbivore choice assays. The experiment included six populations of *Clarkia xantiana* (Onagraceae), which span a complex environmental gradient in the Southern Sierra Nevada of California. *Clarkia xantiana*'s developing fruits are commonly attacked by grasshoppers at the end of the growing season, and the frequency of attack is more common in populations from the range center than range margin. We found that AMF negatively influenced all metrics of plant growth and reproduction across all populations, presumably because plants supplied carbon to AMF but did not benefit substantially from resources potentially supplied by the AMF. The fruits of plants infected with AMF did not differ from those without AMF in their resistance to grasshoppers. There was significant variation among populations in damage from herbivores but did not reflect the center‐to‐margin pattern of herbivory observed in the field. In sum, our results do not support the view that AMF interactions modulate plant–herbivore interactions in this system.

## 1 INTRODUCTION

Organisms in natural ecosystems participate in a web of mutualistic and antagonistic species interactions. For plant species, interactions with herbivores, pathogens, mutualists, and competitors occur both aboveground and belowground, comprising two subsystems that feedback with each other to regulate community structure and ecosystem functioning (e.g., Bardgett, 2005; Van der Putten, Vet, Harvey, & Wäckers, 2001; Wardle, 2002). For example, the interaction between plants, their belowground microbial mutualists, and aboveground insect herbivores can affect the fitness and community dynamics of all three partners (reviewed in Gehring & Bennett, 2009). The outcome of this interaction, however, varies greatly among systems, and the reasons for this variation are not always clear (Gehring & Bennett, 2009).

Arbuscular mycorrhizal fungi (AMF) are belowground plant symbionts that colonize the cortical root cells of vascular plants. The association is common, found in an estimated 80% of vascular plant families, and is characterized by an exchange of soil nutrients and water accessed by AMF in exchange for plant photosynthate. AMF often improve plant establishment, survival, and growth and can also confer tolerance to high concentrations of salts and heavy metals (Hildebrandt, Regvar, & Bothe, 2007; Porcel, Aroca, & Ruiz‐Lozano, 2012; Smith & Read, 2008). Plants inoculated with AMF often also show improved growth under water stress (Augé, 2001). Although this symbiosis is thought to be largely mutualistic in natural populations, the outcome of AMF–plant interactions is often context dependent (Hoeksema et al., 2010; Johnson, Graham, & Smith, 1997; Klironomos, 2003). A mutualistic relationship is expected to occur in environments with low soil nutrients, limited water availability, and/or high light levels (Johnson, Rowland, Corkidi, Egerton‐Warburton, & Allen, 2003; Johnson et al., 1997). However, under extremely arid conditions the symbiosis may fail because fungal hyphae are unable to grow and persist, and the plant conducts minimal photosynthesis to conserve water via stomatal closure (Dosskey, Boersma, & Linderman, 1991).

Because AMF interactions affect plant physiology in such diverse ways, it is no surprise that they can have complex, indirect influences on a plant's interactions with other organisms. AMF have been found to affect interactions with a number of plant mutualists, including pollinators (Gange & Smith, 2005; Wolfe, Husband, & Klironomos, 2005) and rhizobia (Larimer, Clay, & Bever, 2014), as well as plant enemies, such as pathogens (Borowicz, 2001) and herbivores (Gehring & Whitham, 2002). Given the potentially large effects of both herbivory (Maron and Crone 2006) and mycorrhizal fungi (Koide & Dickie, 2002) on plant populations, the tripartite interaction of plants, AMF, and herbivores has received considerable attention. Previous experiments on plant–microbe–herbivore interactions have produced highly variable results (Gehring & Bennett, 2009), with the net outcome depending on factors such as herbivore feeding specialization (Koricheva, Gange, & Jones, 2009) and various abiotic stresses (Pineda, Dicke, Pieterse, & Pozo, 2013). Herbivory may increase with AMF because plants acquire more nutrients and grow more quickly resulting in more abundant and high‐quality food for herbivores (Smith & Read, 2008; Vannette & Hunter, 2011). Alternatively, herbivory may decrease with AMF because of changes in plant defense, either via immune system “priming” due to fungal colonization of plant tissue (Jung, Martinez‐Medina, Lopez‐Raez, & Pozo, 2012), or increases in constitutive or inducible defense compounds (Gange and West 1994, Bennett & Bever, 2009). The magnitude of positive or negative microbial effects on plant resistance to herbivores may be amplified under abiotic stress because of crosstalk between plant signaling pathways induced by each stressor (Pineda et al., 2013).

Plant–AMF–herbivore interactions likely depend on light availability, soil nutrient content, and other environmental factors that influence the rates of resource exchange between plants and AMF and therefore plant tissue quality. Populations of plants may adapt to their local abiotic and/or biotic soil environments in part through their response to AMF colonization (Kaeppler et al. 2000, Cavender & Knee, 2006; Johnson et al., 2010), which may in turn cause population variability in AMF‐driven plant–herbivore interactions. Two studies have examined how plant genotypes differentially respond to AMF, and how this variable response translates into different effects on herbivores, both finding that plant genotype can significantly alter the outcome of this tripartite interaction (Bennett, Millar, Gedrovics, & Karley, 2016; Garrido, Bennett, Fornoni, & Strauss, 2010). However, only one study (Rasmussen et al., 2017) has incorporated geographic variation in studies of plant–AMF–herbivore interactions; this study found variation among three populations of *Plantago lanceolata* in both response to AMF and subsequently, effects on herbivores in feeding trials. While some work has been done looking at plant–microbe–herbivore interactions for species in the process of range expansion (Engelkes et al., 2008), no studies that we know of have sampled plant populations from the center to edge of a species’ current geographic distribution where a strong environmental gradient might contribute to variation in the outcome of species interactions.

In this study, we examined multispecies interactions among plants, AMF, and herbivores using six populations of the annual plant *Clarkia xantiana* ssp. *xantiana* (hereafter, *xantiana*). *Xantiana* is colonized by AMF in the field and experiences a strong geographic gradient in the strength of its interactions with insect herbivores, especially grasshoppers. Damage by grasshoppers occurs principally on developing fruits and seed loss to herbivores can exceed 40% in some populations (D. Moeller, unpublished data). The severity of insect herbivory declines considerably from center to margin of *xantiana*'s geographic distribution, and resistance to herbivory within populations reflects this geographic trend, with higher herbivore resistance observed in central populations (D. Moeller, unpublished data). This variation in herbivory occurs across a marked gradient in abiotic factors, where precipitation becomes lower and less predictable toward the range edge (Eckhart et al., 2011). With the complex gradient in abiotic and biotic factors across *xantiana*'s range, the outcome of interactions between plants, microbial symbionts, and insect herbivores may also change across geographic space and among plant populations.

Here, we examine how AMF and plant source population interact to affect plant performance and the severity of herbivory under water stress using a greenhouse experiment. Specifically, we ask the following questions:

1. Does AMF inoculation affect *xantiana* growth and phenology under low water availability?
2. Does AMF inoculation alter herbivore preference for *xantiana* fruits?
3. Does this tripartite interaction vary among plant populations spanning the range of the subspecies?

## 2 MATERIALS AND METHODS

### 2.1 Natural history of *Clarkia xantiana*

*Clarkia xantiana* ssp. *xantiana* is a primarily outcrossing winter annual forb endemic to southern California. It occurs primarily on steep, sandy slopes in the southern Sierra Nevada at elevations of 500–1500 m (Eckhart & Geber, 1999; Lewis & Lewis, 1955). Seeds germinate in November–December, and plants mature in May–June. The populations used in this experiment are distributed along a strong environmental gradient from the center of *xantiana*'s distribution to its eastern range edge, with mean annual precipitation and temperature decreasing, and variability in precipitation increasing, as populations approach the eastern range limit (Eckhart et al. 2010, Eckhart et al., 2011). Soils in the center of *xantiana*'s range are mostly derived from igneous rock, with metasedimentary soils becoming increasingly frequent at the eastern range edge (Eckhart et al. 2010). One population used in this study, S22, occurs on soils derived from metasedimentary rock; all others occur on igneous soils.

*Xantiana* plants are attacked by multiple herbivores. Fatal mammalian herbivory becomes increasingly frequent toward the eastern range edge (J. Benning, unpublished data), while invertebrate herbivory is highest in central populations (D. Moeller, unpublished data). Defoliation by larvae of *Hyles lineata* (White‐lined Sphinx Moth) occurs occasionally (personal observation), but the most common invertebrate herbivory is by grasshoppers (*Melanoplus* sp.). These generalist herbivores feed on immature fruits of *xantiana* by eating the immature, green pericarp and/or immature seeds; this fruit herbivory can have significant fitness consequences for both individuals and populations of *xantiana*, with some populations losing more than 40% of their fruits on average to grasshopper herbivory (D. Moeller, unpublished data).

Surveys in natural populations show that the subspecies is colonized by AMF in the field (J. Benning, pers. observation).

### 2.2 Experimental design

**Figure 1:**
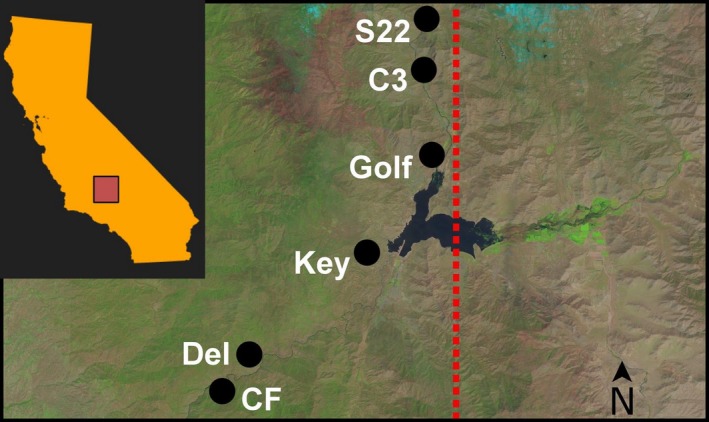
Map showing locations of six populations of *Clarkia xantiana* ssp*. xantiana* used in this experiment. Dashed red line marks eastern geographic range limit of *C. x. xantiana*. Inset shows population sampling area within California, USA. LIDAR photograph taken 22 April 2017, showing west‐to‐east gradient of increasing aridity

We conducted a greenhouse experiment in St. Paul, Minnesota, USA manipulating AMF inoculation and source population of *xantiana* in a 2 × 6 full factorial design (*n* = 50 plants per treatment combination, 600 plants total). We applied each of two AMF treatments—AMF‐inoculated (M+) and AMF‐free (M−)—to half of the individuals from each population. In order to capture variation in *xantiana*'s AMF responses and herbivore resistance, we chose source populations that span from the center to edge of its range (Figure 1)*;* the source populations used were (from center to edge): CF (109x, 35°00′94″E), Del (107x, 35°31′27″E), Key (57x, 36°28′64″E), S22 (22x, 36°90′76″E), C3 (21x, 36°91′07″E), and Golf (3x, 37°05′82″E). (Population labels with “x” are included here to correspond to earlier published work with *xantiana*, but we use the preceding labels throughout the manuscript for simplicity.) We used field‐collected seeds from 10 maternal families within each population. All seeds were collected from the field during the summer of 2015.

*Xantiana* experiences low nitrogen availability in the field (field NO_3_ mean ± *SD*: 4.26 ± 4.94 ppm, averaged across five of the six populations used in this experiment), and we prepared our soil mix (2:1 sand (Fischer Mining Co., MN, USA): growing mix (SunGro LC8; SunGro, MA, USA); this growing mix typically contains 50–140 ppm NO_3_) to reflect the low nutrient charge of natural soils. The soil mixture was steam pasteurized for 2 hr at 80°C and then allowed to cool for at least 2 days before planting. We prepared 600 1 L pots (Stuewe & Sons D60 Deepots) for planting by submerging in 0.9% sodium hypochlorite (bleach) solution, and lining the pot bottoms with newspaper.

Arbuscular mycorrhizal fungi treatments were applied to the soil mixture prior to planting. Plants receiving the M+ treatment were planted into pots with a 25‐ml layer of Micronized Endomycorrhizal Inoculant (BioOrganics Inc, New Hope, PA, USA) spread ca. four inches below the soil surface This commercial AMF inoculum contains a blend of nine endomycorrhizal species (*Glomus aggregatum, G. etunicatum, G. clarum, G. deserticola, G. intraradices, G. monosporus, G. mosseae, Gigaspora margarita,* and *Paraglomus brasilianum*) and has been used successfully to induce AMF colonization in a wide range of plant species (Babikova et al., 2014; Berg, Eaton, & Ayres, 2001; Cavender & Knee, 2006; Pischl & Barber, 2016; Wiseman & Wells, 2009). The non‐mycorrhizal control treatment (M−) pots only contained 2:1 sand:growing mix. Two seeds were then sown on top of the soil.

Pots were placed in a cold room with 24‐hr light (2–150 W bulbs) at 8°C for the first 6 days, then 12°C for six more days. While in the cold room, plants were misted daily. After 12 days in the cold room, and 3 days after the first germinants were observed, pots were moved to the greenhouse. Fourteen pots had no germination; for those pots, pre‐germinated seeds from the assigned population and maternal family were sown on top of the soil.

In the greenhouse, plants were maintained at ~25 to 27°C for the first month on a 14‐hr/10‐hr L/D cycle and then ~23 to 25°C for the remainder of the experiment on the same L/D cycle. For the first 6 weeks in the greenhouse, plants were watered regularly to ensure early survival. They then received four waterings 2 weeks apart, and then, watering was stopped altogether for the final 7 weeks of the experiment. This watering regime was designed to mimic the seasonal conditions experienced by *xantiana* in the field, as rainfall tapers off and eventually stops altogether toward the end of their growing season. Survival was very high, with 95%–99% of individuals (of 100) surviving for each population. Sample sizes for Key were lower (59) because field collections mistakenly included some individuals of *xantiana*'s sister subspecies, *C. xantiana* ssp. *parviflora*, as these are difficult to distinguish in the field after fruit set. Those *parviflora* individuals were excluded from analysis (this did not skew sample sizes between AMF treatments for Key: M+ *n* = 29; M− *n* = 30).

Once plants began flowering, we generated fruits by crossing random pairs of plants from the same population × AMF treatment, but from different maternal families. Two to six flowers per plant were crossed.

We measured two phenological traits—days to first flower and time spent flowering—and four growth allocation metrics—number of flowers produced, as a proxy for reproductive effort; number of seeds per fruit; average seed weight; and shoot dry biomass. Days to first flower were recorded as the number of days between planting and the day the first flower opened, and time spent flowering was the number of days between the day the first flower opened and the day the last flower senesced. All hand‐pollinated fruits not used in herbivore trials were collected at maturity; for each of these fruits, the number of seeds per fruit was counted, and the mass of all seeds from a fruit was quantified. Individual seed weight was calculated by dividing total seed mass from a fruit by the number of seeds in that fruit. After plants had senesced, their stems were clipped at the soil level and desiccated in a drying oven at 55°C prior to measuring shoot dry biomass. All stems were collected over the course of 7 days.

### 2.3 Herbivore choice trials

We conducted herbivore choice trials on a subset of fruits to assess whether AMF may confer herbivore resistance to host plants, and how those effects may vary among source populations. Our trials occurred only using fruits, which avoids the potentially confounding effects of fruit number, plant architecture, or plant size when using whole plants in herbivory trials. We used young adults of the generalist grasshopper, *Melanoplus sanguinipes* (USDA ARS Northern Plains Agricultural Research Laboratory), as the herbivore in our feeding trials. *Melanoplus devastator*, a close relative, is a common herbivore of *xantiana* in the field. It is unclear whether *M. devastator* and *M. sanguinipes* are different taxa because the main differentiating feature is geographic location, whereas other attributes do not appear to differ (Dingle & Mousseau, 1994; Orr, Porter, Mousseau, & Dingle, 1993). Grasshoppers were maintained in ventilated plastic tubes kept in growth chambers at 30°C, 16:8 L/D. They were fed a diet of organic romaine lettuce and Cheerios (General Mills, Minneapolis, MN) and were thus naïve to variation in *xantiana* tissue quality.

**Figure 2:**
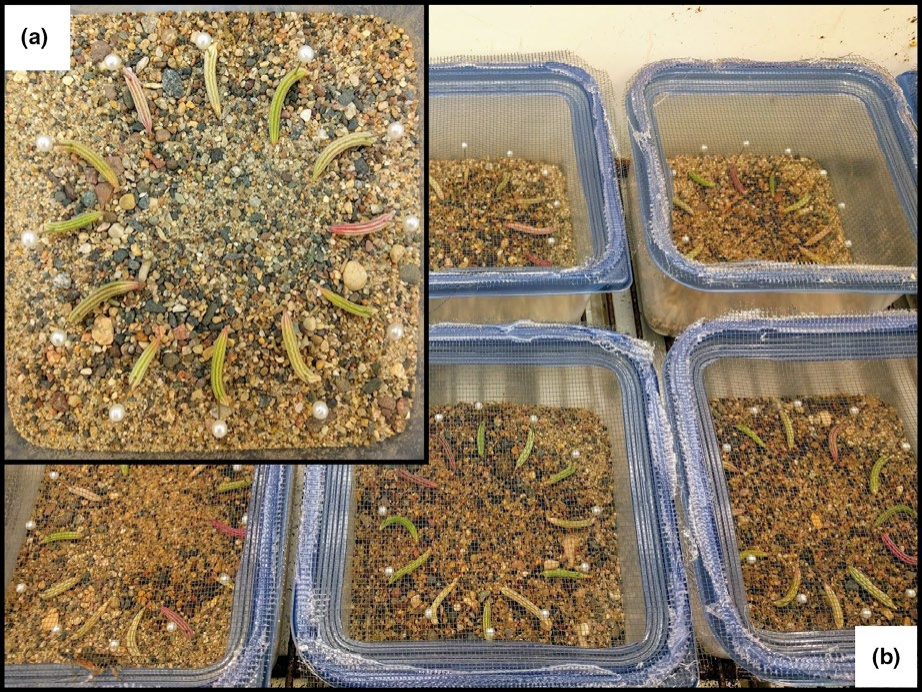
Inset (a) shows an individual feeding preference trial, with fruits from each population × AMF treatment arranged in a circle within a plastic Ziploc container. (b) shows a group of these trials, each containing one grasshopper, arranged in the growth chamber

Trial arenas were constructed in plastic Ziploc containers (15.5 × 15.5 × 8.5 cm) with a square piece of medium‐density fiberboard placed in the bottom. We included one fruit from each population × AMF treatment in every trial, for a total of 12 fruits per trial. We drilled 12 holes equidistantly around the perimeter of a circle in the MDF board, and each fruit was pinned into a randomly assigned hole. The MDF board was then covered with sand to mimic field conditions, with the fruits sitting just above the level of the sand, and the tops of the Ziploc containers were replaced with fine mesh to allow air movement (Figure 2). Prior to trials, all fruits were sprayed with water to delay desiccation.

On consecutive days, we conducted two rounds of choice trials which included 20 and 12 trial arenas, respectively. One trial in round two was discarded due to the death of the grasshopper during the trial, resulting in a total of 31 choice trials. Two of the seven grasshopper tubes were randomly selected for each round, before which grasshoppers in those tubes were starved for 27.5 hr. Grasshoppers were then haphazardly chosen from the tubes for trial. One grasshopper was placed into each arena and allowed to feed on *xantiana* fruits for 17.5 hr. In addition, round one had two control trials, which received no grasshopper, and round two had one control trial. Trials were conducted at 32°C, 16:8 L/D.

We assessed the severity of herbivory using a categorical scoring system. Grasshoppers produce two distinct types of herbivory on *xantiana* fruits, which we called ”scraping” and “chomping.” “Scraping” herbivory appears as rasping on the side of a fruit that may or may not breach the ovary wall; “chomping” is a shortening of the fruit from the tip toward the peduncle, and usually results in a greater fitness loss (from devouring of immature seeds) than “scraping”. Fruits used in herbivory trials were scored on a scale of 0–3 based on the type of herbivory they suffered. A fruit was scored “0” for no/trace herbivory; “1” for 10%–50% scraping; “2” for 50%–100% scraping; or “3” for chomping. A binary measure of herbivory was also used in analysis, for which herbivory scores 1–3 were condensed into the single factor “herbivory present,” and 0 scores were “herbivory absent.” Herbivory scoring was “blind”; that is, the herbivory scorer was unaware of which treatment combination each fruit came from while scoring.

### 2.4 Statistical analyses

We used linear mixed models to test for the effects of plant population and AMF inoculation on plant fitness proxies (biomass and flower number), phenology (time to begin flowering and time spent flowering), and seed production (seeds per fruit and seed weight). For fruits that were hand pollinated, seeds per fruit was calculated for each plant as the mean number of seeds per fruit, with the number of analyzed fruits on a plant ranging from one to six. Some fruits that were hand pollinated did not fill any seeds, which most likely reflected unsuccessful hand pollination, rather than abortion due to a lack of resources. Consequently, we removed seedless fruits from seed analyses. Seed weight was calculated for each plant as the mean weight of a single seed. We included all plants with at least one fruit that produced two or more seeds (*n* = 423).

Treatment effects were examined using a type‐II ANOVA using the Afex package in R (R Core Team 2017). Effect sizes were compared between the Kenward–Rogers and Satterthwaite approximations for denominator degrees of freedom; minimal differences were found between these methods, so Kenward–Rogers values are reported. Days to first flower, time spent flowering, dry biomass, number of flowers produced, number of seeds per fruit, seed weight, and seed weight per fruit were included separately as response variables. Population, AMF treatment, and population x AMF interaction were included as fixed effects, and maternal family (nested within population) was included as a random effect. Normality and homoscedasticity of residuals were verified visually. For models that showed variation in response among populations, Tukey's post hoc test was employed to detect pairwise differences between populations using the Holm correction for multiple comparisons.

Although the inclusion of multiple maternal families was primarily to capture a representative sample of population genetic variation, we also explored the potential for differences in the direction of response to mycorrhizal fungi (positive versus negative) among maternal families. To do this, we performed t tests comparing biomass and reproductive effort of M+ and M− plants for each family. If the 95% CI of the difference between M+ and M− groups did not span zero, we interpreted the family as having a significantly positive or negative response.

We tested for an effect of population and AMF treatment on herbivory levels using both a cumulative link mixed model (CLMM) and a generalized linear mixed model (GLMM). In the CLMM, herbivory score (0–3) was treated as an ordinal categorical response variable; in the GLMM, presence/absence of any herbivory (including both scraping and chomping) was included as a binomial response variable. Population, AMF treatment, and population × AMF interaction were included as fixed effects; trial and maternal family were included as random effects. For CLMM and GLMM models, significance of fixed factors was determined through likelihood ratio tests; nonsignificant factors were dropped from both models. Subsequent pairwise comparisons of factor levels were tested using Tukey's contrasts at **α** = 0.05 in the lsmeans package in R. All analyses were conducted using the R statistical platform (R Core Team 2015).

## 3 RESULTS

### 3.1 Plant responses

**Figure 3:**
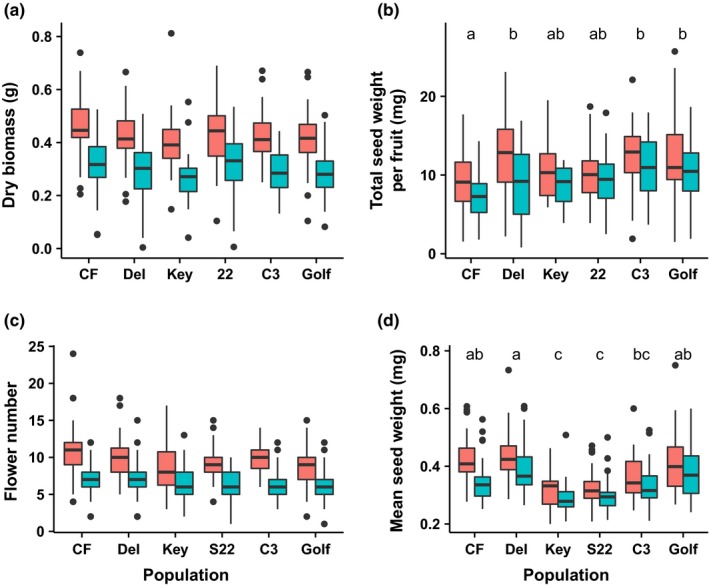
Growth and reproductive effort metrics for AMF‐inoculated (blue) and AMF‐free (red) plants sourced from populations across the range of *xantiana*. Populations are ordered across the *x*‐axis from range center to edge. For all panels, AMF treatment is significant at *p *< 0.001. Different letters above populations indicate significant differences according to Tukey's pairwise comparison of least square means and are indicated only for response variables showing a significant population effect

Mycorrhizae significantly reduced plant biomass (*F*_1,488.2_ = 237.5, *p* < 0.001), flower production (*F*_1,486.1_ = 231.4, *P *< 0.001), seed mass (*F*_1,371.4_ = 34.5, *p* < 0.001), and seed weight per fruit (*F*_1,376.6_ **= 22.3, *p *< 0.001) by 30%, 32%, 10%, and 15%, respectively (Figure 3; Supporting information Table S1). They also produced 6% fewer seeds per fruit although the difference was marginally nonsignificant (*F*_1, 384.3_ **= 3.0, *p* = 0.08). Total seed weight per fruit was significantly lower in CF plants than Del, C3, or Golf (Figure 3b). Seeds from Key and S22 were significantly lighter than all other populations except C3 (Figure 3d). Populations did not differ in their growth and seed production responses to AMF inoculation (Population × AMF term for responses in the order they were mentioned: *F*_5,488.8_ = 0.29, *p* = 0.92; *F*_5,485.8_ = 1.12, *p* = 0.35; *F*_5,371.2_ = 1.14, *p* = 0.34; *F*_5,376.2_ = 0.64, *p* = 0.67; *F*_5,376.4_ = 0.36, *p* = 0.87). There were no significant differences among maternal families in direction of response to AMF inoculation for either biomass or reproductive effort (Supporting information Figure S1).

**Figure 4:**
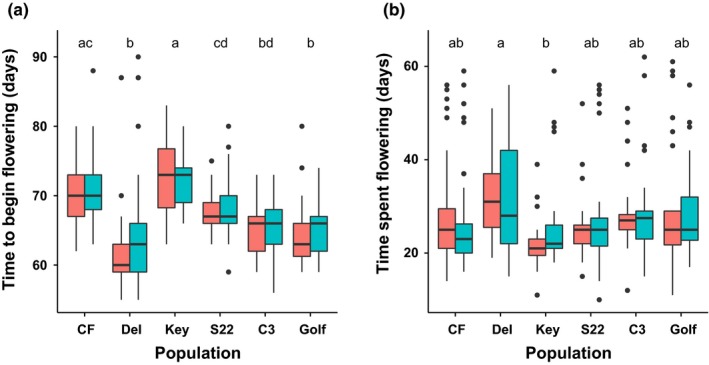
Phenology of AMF‐inoculated (blue) and AMF‐free (red) plants sourced from populations across the range of *xantiana*. Populations are ordered across the *x*‐axis from range center to edge. Different letters above populations indicate significant differences according to Tukey's pairwise comparison of least square means

Individual plants initiated flowering over a 10‐day period. Overall, AMF inoculation significantly delayed flowering by 1 day (*F*_1,480.5_ = 6.6, *p *< 0.05; Figure 4a). The largest delay in flowering was observed in the earliest flowering population, Del, where AMF inoculation delayed flowering by 3 days (Tukey's adjusted *p* = 0.02). Plant populations showed significant variation in time to flowering, although there was no clear geographic pattern (Figure 4a). The only significant population difference in flowering duration was between Del (31.4 ± 2.9 days) and Key (24.2 ± 3.5 days; Tukey's adjusted *p* = 0.03; Figure 4b). There was no effect of AMF on flowering duration.

### 3.2 Herbivore responses

AMF inoculation did not affect herbivore preference (CLMM: LR = 4.1, *df *= 6, *p* = 0.66; GLMM: LR = 3.1, *df* = 6, *p* = 0.8), suggesting that AMF infection did not confer greater plant resistance or susceptibility to herbivores. The overall fraction of fruits damaged by herbivores in trials was 56%. Of the damaged fruits, 27% received chomping herbivory and 73% received scraping herbivory.

**Figure 5:**
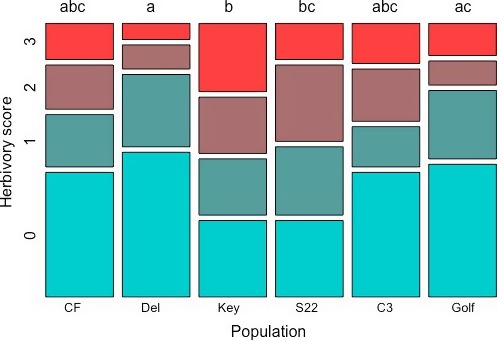
Variation in herbivory damage among source populations in herbivore choice trials. Populations are ordered across the *x*‐axis from range center to edge. Severity of herbivory increases with herbivory score (see Materials and Methods). Different letters above populations indicate significant differences according to Tukey's pairwise comparison of least square means with a significance level of α* *= 0.05

Since likelihood ratio tests indicated no effect of AMF on herbivory rates, we continued analyses with the reduced model, testing the effect of plant population on herbivory, with trial and maternal family included as random factors. Populations varied significantly in herbivory, with the CLMM (accounting for the severity of herbivory) and GLMM (with herbivory as a binary response variable) models resulting in similar estimates of population differences. Overall, Key and S22 suffered the highest rates of herbivory, and Del and Golf suffered the lowest rates of herbivory in herbivore choice trials. Pairwise comparisons of populations for both GLMM and CLMM indicated significantly more herbivory for Key and S22 than for Del, and CLMM additionally identified Key as experiencing significantly more intense herbivory than Golf (Figure 5). Least‐squares means from GLMM indicated that fruits from Del and Golf plants had 43% and 48% probability of herbivory, respectively, while fruits from Key and S22 plants each had 72% probability of herbivory. CLMM results indicated similar trends, with a tendency toward more severe herbivory in Key (proportional odds = 2.4, *p* = 0.02) and S22 (proportional odds = 1.8, *p* = 0.1).

## 4 DISCUSSION

### 4.1 Herbivore responses to AMF and plant population

AMF interactions with *xantiana* consistently and negatively affected plant performance, yet had no measurable effects on patterns of herbivory by the most destructive insect herbivore in natural populations (grasshoppers; *Melanoplus* spp.). The net effect of AMF on herbivory is likely conditional upon how plants respond to AMF in terms of tissue nutrient concentration, defensive chemical concentration, and growth (Gehring & Whitham, 2002; Koricheva et al., 2009). Therefore, the outcome of these aboveground–belowground interactions is complex and context dependent. For example, a review by Gehring and Bennett (2009) reported that 45% of studies investigating the effects of AMF on insect herbivory (from 20 publications) showed a net increase, 35% showed a net decrease, and 21% showed no significant effect. Decreases in herbivory due to AMF may be due to increased accumulation of constitutive defenses in plant parts or because AMF colonization of root cortical cells “primes” the plant immune system. In the latter case, AMF colonization provokes jasmonic acid‐based plant immune responses, such that plants can upregulate the production of defense compounds more rapidly upon herbivore attack (Jung et al., 2012). Our study involved the harvest of fruits from plants that experienced no damage prior to herbivory trials and therefore is best suited for evaluating whether AMF influence herbivory via constitutive defense production.

Previous studies have shown that AMF cause greater constitutive defense production in some plant species, which in turn causes reduced herbivory; however, the results are mixed. For example, Dingle and Mousseau (1994) found increased levels of aucubin and catalpol, carbon‐based herbivore deterrent compounds, in leaves of AMF‐colonized *Plantago laureolata* plants compared to fungicided controls. They reasoned that the change in abundance of these compounds was due to altered carbon/nutrient balance in mycorrhizal plants, but herbivory trials with two different insects showed contrasting results—chewing insects (caterpillars) favored the non‐mycorrhizal plants but sucking insects (aphids) showed no preference (see also Roger et al. 2013). Another recent study found that AMF inoculated plants did not suffer significantly different levels of herbivory than control plants (Barber, Kiers, Hazzard, & Adler, 2013), despite differences in leaf nutrient concentrations between treatments. Our results, showing no influence of AMF inoculation on herbivore preference, reflect the conclusions of a recent review (Gehring & Bennett, 2009) that found effects of mycorrhizal interactions on plant–herbivore dynamics vary widely and are highly context dependent. In a meta‐analysis of plant–mycorrhizae–herbivore studies (including both ecto‐ and endomycorrhizal experiments), Koricheva et al. (2009) came to similar conclusion of high variability and context dependency in these aboveground–belowground interactions, but did find a trend of decreased herbivore performance on AMF plants when the herbivore was a polyphagous chewing insect (such as the grasshoppers in this study), but not when the herbivore had a more restricted diet or was a sucking insect. Our results do not fit this pattern, although we did not measure herbivore performance, but rather herbivore preference.

Herbivores did respond significantly differently to *xantiana* populations where two populations (Key, S22) experienced greater damage than the remaining populations. Those populations (Key, S22) occur near or at the range margin; however, the other marginal populations do not exhibit comparably high levels of herbivory (C3, Golf). Therefore, we did not observe a convincing geographic pattern to herbivore preference as we might have predicted based upon field studies that documented greater herbivore resistance in central populations.

### 4.2 Plant responses to AMF

We found that all aspects of growth and reproductive effort were reduced in AMF inoculated plants, suggesting that the net effect of AMF inoculation on *xantiana* was parasitic in our experiment. This relationship did not vary among *xantiana* populations, as evidenced by a nonsignificant interaction between AMF and population treatments. Symbioses occur on a parasitism mutualism continuum, with the outcome of the symbiosis depending on environmental factors such as nutrient and light availability, and the genotypic identities of the plants and the AMF (Johnson et al., 1997; Klironomos, 2003). A meta‐analysis by Hoeksema et al. (2010) found overall positive growth effects in response to mycorrhizal inoculation (both AMF and ectomycorrhizal fungi with non‐N‐fixing forbs), especially when multiple fungal species were used as inoculum. However, adverse growth effects on forbs in response to AMF are not uncommon. For example, in multiple field and greenhouse studies of agricultural tobacco, *Glomus macrocarpum* consistently and significantly reduced tobacco root length, aboveground biomass, and reproductive effort (Hendrix, Jones, & Nesmith, 1992; Jones & Hendrix, 1987; Modjo & Hendrix, 1986; Modjo, Hendrix, & Nesmith, 1987). Likewise, in a study testing outcomes of many pairwise combinations of plant and AMF species, Klironomos (2003) found large variation in plant growth responses to AMF inoculation, with AMF having both strongly negative and strongly positive effects on plant biomass. In that study, plant growth response ranged from −49% to +46% (% difference in plant growth between AMF and non‐AMF plants). In light of these previous results, the AMF effects in the current study (−30% difference) fall within the range previously observed and toward the negative end of the spectrum. Further investigation may reveal whether the adverse growth effects witnessed in the current study were due to environmental aspects of the greenhouse treatments, the combination of *xantiana* and AMF genotypes, or both.

This experiment showed consistent, negative effects of AMF inoculation on not only plant growth, but other performance metrics more directly tied to individual lifetime fitness. Biomass is used as a fitness proxy in most experiments testing effects of AMF (Hoeksema et al., 2010), but in this study we also documented significant reductions in flower number and seed weight, and a marginally significant reduction in seed number, for AMF‐inoculated plants. This highlights the fact that reductions in growth due to parasitic AMF interactions likely will be realized as significant reductions in lifetime fitness, even though direct measure of individual plants’ seed output is rarely reported in the AMF literature.

Low soil nitrogen availability has been shown to shift the mycorrhizal symbiosis toward parasitism in other systems (Johnson, Wilson, Wilson, Miller, & Bowker, 2015), and the low nutrient charge of the soil mix in this experiment, chosen to reflect field soil conditions (Methods), could have contributed to the parasitic effects of the AMF inoculum. Additionally, if there were microbial species in the inoculum other than AMF, they could have contributed to the plant response observed. The media of AMF inoculum can also change abiotic soil properties within inoculated pots, such as by increasing soil nutrient availability (Rowe, Brown, & Claassen, 2007), but any positive effects of nutrient addition via inoculum media were outweighed by negative effects of AMF inoculation in this experiment.

Although we saw this community of nine AMF species have adverse effects on *xantiana* in the greenhouse, it remains to be seen what effect AMF have on *xantiana* in a field setting, and how locally sourced AMF species affect growth. Most studies using commercially produced inocula reported increased plant growth in inoculated plants compared to uninoculated plants (Baum, El‐Tohamy, & Gruda, 2015), including some that found commercial inoculum to provide benefits similar to or greater than native inoculum (Barber et al., 2013; but see Rowe et al., 2007; White, Tallaksen, & Charvat, 2008). Nevertheless, there is some evidence of genotype × genotype interactions between plants and root endophytes (Johnson et al., 2010), which suggests that local adaptation to microbial communities may influence the outcome of interactions. Greenhouse conditions have also been implicated in studies reporting AMF parasitism, since low levels of light during winter months may sufficiently depress photosynthetic rates that the nutrient benefit provided by AMF is not enough to overcome the carbon cost. Finally, field plants experience a complex biotic community that is absent from the greenhouse, and it is possible that positive effects of AMF are only realized in this more complex biotic context, where plants are interacting with multiple other microbial and invertebrate species (Hoeksema et al., 2010).

In addition to affecting growth and reproductive effort, microbial communities have also been shown to affect plant fitness by altering plant phenology. In arid regions like those inhabited by *xantiana*, plants can avoid periods of low water availability late in the growing season by shifting phenology and flowering earlier (Aronson, Kigel, Shmida, & Klein, 1992; Volis, 2007). For example, Lau and Lennon (2012) showed that the microbial community of the annual plant, *Brassica rapa*, was responsible for a phenological shift to earlier flowering under drought conditions. We found that, overall, AMF inoculation delayed flowering of *xantiana* by 1 day. This AMF‐induced phenological shift was stronger in one population, Del, where flowering was delayed by 3 days with AMF inoculation (all plants initiated flowering within a 10‐day time period). This delay may be due to direct influence of AMF colonization on biochemical pathways influencing the switch from growth to reproduction in *xantiana*, or an indirect effect where plant resource allocation to AMF hinders development and delays flowering. Earlier flowering time in *xantiana* is favored by selection in some environments (Geber and Eckhart 2005), in part because flowering time strongly influences a plant's probability of fatal mammal herbivory in some parts of *xantiana’*s range. For example, at the edge of *xantiana*'s range, each day delay in flowering increases a plant's odds of fatal herbivory by 5%, and this fatal mammal herbivory can have large effects on population growth rates (Benning, Eckhart, Geber, & Moeller, 2018). Our results suggest that microbial communities may play an important role in modulating the expression of this ecologically important trait.

## 5 CONCLUSIONS

Plant–AMF interactions have been shown to indirectly affect plant–herbivore interactions by influencing the quantity and quality of plant tissues as well as the production of defensive compounds (Smith and Read 1997, Bennett, Alers‐Garcia, & Bever, 2006). In this study, we found consistent negative effects of a community of nine AMF species on plant growth and reproduction but no indirect effects on herbivory to fruits by insect herbivores. The negative effect of AMF on plant growth does not appear to similarly influence plant tissue quality from the perspective of grasshoppers. Populations sampled from across a geographic gradient in the abiotic and biotic environment did not differ in plant responses to the AMF community. There was some variation in herbivore preference for fruits from different plant populations, but not a clear geographic pattern. Overall, our results are consistent with previous observations that plant–AMF interactions vary considerably from mutualistic to parasitic, and that plant–herbivore interactions may not be modified by AMF even when AMF effects on plants are strong.

## AUTHOR CONTRIBUTIONS

LB, JB, and DM conceived the ideas and methodology. LB wrote the manuscript; LB and JB collected and analyzed the experimental data. All authors contributed critically to the drafts and gave final approval for publication.

## DATA ACCESSIBILITY STATEMENT

All experimental data are available at datadryad.org: https://doi.org/10.5061/dryad.176gv58.
