# Supplementary material for: Mycorrhizal interactions do not influence plant–herbivore interactions in populations of Clarkia xantiana ssp. xantiana spanning from center to margin of the geographic range
Source: Ecol Evol. 2018 Oct 26;8(22):10743–53. doi: 10.1002/ece3.4523 (PMC6262727; doi:10.1002/ece3.4523)
Supplement: Supplementary file 1 [file ECE3-8-10743-s001.docx]

**Title**

Mycorrhizal interactions do not influence plant-herbivore interactions in populations of *Clarkia xantiana* ssp. *xantiana* spanning from center to margin of the geographic range

**Authors**

Bolin, L.G.; Benning, J.W.; Moeller, D.M.

**Supporting Information**

**Table S1**. Type II ANOVA table for linear mixed models

| *Response* | *Term* | *df* | *F* or *χ^2^* | *P* |
| --- | --- | --- | --- | --- |
| **Biomass** | Population | 5, 48.9 | 5.2 | <0.001 *** |
|  | AMF | 1, 376.6 | 22.3 | <0.001 *** |
|  | Population x AMF | 5, 376.2 | 0.6 | 0.672 |
|  | Maternal Family | 1 | 9.7 | 0.002 ** |
| **Flower Number** | Population | 5, 49.8 | 2.2 | 0.071 **^†^** |
|  | AMF | 1, 486.1 | 231.4 | <0.001 *** |
|  | Population x AMF | 5, 485.8 | 1.1 | 0.349 |
|  | Maternal Family | 1 | 18.0 | <0.001 *** |
| **Days to First Flower** | Population | 5, 49.8 | 18.5 | <0.001 *** |
|  | AMF | 1, 480.7 | 6.6 | 0.010 * |
|  | Population x AMF | 5, 480.5 | 1.9 | 0.087 **^†^** |
|  | Maternal Family | 1 | 9.1 | 0.003 ** |
| **Days Spent Flowering** | Population | 5, 49.0 | 2.2 | 0.055 **^†^** |
|  | AMF | 1, 430.7 | 0.5 | 0.469 |
|  | Population x AMF | 5, 431.2 | 0.9 | 0.467 |
|  | Maternal Family | 1 | 34.7 | <0.001 *** |
| **Number of Seeds Per Fruit** | Population | 5, 48.9 | 6.7 | <0.001 *** |
|  | AMF | 1, 376.8 | 3.0 | 0.084 **^†^** |
|  | Population x AMF | 5, 376.4 | 0.4 | 0.873 |
|  | Maternal Family | 1 | 3.5 | 0.063 **^†^** |
| **Seed Weight** | Population | 5, 49.5 | 10.3 | <0.001 *** |
|  | AMF | 1, 371.4 | 34.5 | <0.001 *** |
|  | Population x AMF | 5, 371.2 | 1.1 | 0.34 |
|  | Maternal Family | 1 | 3.5 | 0.061 **^†^** |
| **Seed Weight Per Fruit** | Population | 5, 48.9 | 5.2 | 0.001 *** |
|  | AMF | 1, 376.6 | 22.3 | <0.001 *** |
|  | Population x AMF | 5, 376.2 | 0.6 | 0.672 |
|  | Maternal Family | 1 | 26.2 | <0.001 *** |

All effects are fixed except for Maternal Family. For fixed effects the test statistic F is reported, and degrees of freedom are calculated using the Kenward-Rogers approximation. For the random effect the test statistic *χ^2^* is reported, calculated as twice the difference between log likelihoods of the full model and the model with the random factor excluded.

***, **, *, and **^†^** represent *P* values lower than 0.001, 0.01, 0.05, and 0.1, respectively.


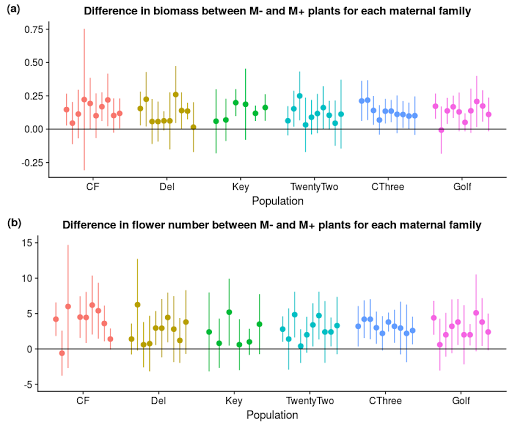


**Figure S1.** Points represent differences in biomass (a) and flower number (b) between M- and M+ plants within each maternal family of each population; line ranges indicate 95% CI from Student’s t-test of M- and M+ plant biomass (a) and flower number (b) for each maternal family in each population. Positive values indicate M- plants in that maternal family had higher biomass (a) or flower number (b) than M+ plants.
